# Streptococcal toxic-shock syndrome due to *Streptococcus dysgalactiae* *subspecies equisimilis* in breast cancer-related lymphedema: a case report

**DOI:** 10.1186/s13256-017-1350-z

**Published:** 2017-07-14

**Authors:** Makoto Sumazaki, Fumi Saito, Hideaki Ogata, Miho Yoshida, Yorichika Kubota, Syunsuke Magoshi, Hironori Kaneko

**Affiliations:** Department of Surgery, Division of Breast and Endocrine Surgery (Omori), Toho University School of Medicine, 6-11-1 Omori-Nishi, Ota-ku, Tokyo, 143-8451 Japan

**Keywords:** Breast cancer, Lymphedema, *Streptococcus dysgalactiaesubspeciesequisimilis*, Streptococcal toxic shock syndrome

## Abstract

**Background:**

Breast cancer-related lymphedema often causes cellulitis and is one of the most common complications after breast cancer surgery. Streptococci are the major pathogens underlying such cellulitis. Among the streptococci, the importance of the Lancefield groups C and G is underappreciated; most cases involve *Streptococcus dysgalactiae* *subspecies* *equisimilis*. Despite having a relatively weak toxicity compared with group A streptococci, *Streptococcus dysgalactiae* *subspecies* *equisimilis* is associated with a mortality rate that is as high as that of group A streptococci in cases of invasive infection because *Streptococcus dysgalactiae* *subspecies* *equisimilis* mainly affects elderly individuals who already have various comorbidities.

**Case presentation:**

An 83-year-old Japanese woman with breast cancer-related lymphedema in her left upper limb was referred to our hospital with high fever and acute pain with erythema in her left arm. She showed septic shock with disseminated intravascular coagulation. Blood culture showed positive results for *Streptococcus dysgalactiae* *subspecies* *equisimilis*, confirming a diagnosis of streptococcal toxic-shock syndrome. She survived after successful intensive care.

**Conclusions:**

To the best of our knowledge, this case represents the first report of *Streptococcus dysgalactiae* *subspecies* *equisimilis*-induced streptococcal toxic-shock syndrome in a patient with breast cancer-related lymphedema. Breast cancer-related lymphedema is a common problem, and we must pay attention to invasive streptococcal soft tissue infections, particularly in elderly patients with chronic disease.

## Background

Breast cancer is one of the most common cancers in women. While survival after breast cancer has significantly improved with the advent of multimodal therapy [1], aggressive treatment sometimes induces complications. In particular, breast cancer-related lymphedema (BCRL) is one of the most common problems, often resulting in the development of cellulitis. Most such cases of cellulitis are caused by streptococci or staphylococci, which are normal bacterial flora of the skin. To date, the streptococcal pathogens causing skin and soft tissue infections have mainly been identified as group A streptococci (GAS), mostly isolated as *Streptococcus pyogenes*. However, other types of β-hemolytic streptococci become more important with ageing. *Streptococcus dysgalactiae* *subspecies* *equisimilis* (SDSE), classified as a group C or G *Streptococcus*, sometimes shows similar clinical manifestations to GAS, but with weaker toxicity. Increased survival of adults with underlying chronic conditions may be a factor contributing to the increasing incidence of SDSE infections [2].

## Case presentation

Our patient was an 83-year-old Japanese woman who had undergone left mastectomy for breast cancer 18 months earlier. The pathological diagnosis according to the Union for International Cancer Control 7th TNM classification for breast cancer was T2 N2 M0. The receptor expression was positive for estrogen and progesterone receptors but negative for human epidermal growth receptor 2. She received adjuvant chemotherapy consisting of four cycles of doxorubicin and cyclophosphamide (AC) and radiotherapy to her axillary and supraclavicular regions. After these therapies, she had been prescribed letrozole. No other relevant past history was found, including no history of diabetes, heart disease, or use of trastuzumab as pharmacotherapy, potentially causing cardiac toxicity. The dose of doxorubicin was lower than cardiac toxicity doses, and there were no abnormalities in organ function. Six months after mastectomy, BCRL of her left upper limb appeared and gradually worsened.

**Fig. 1:**
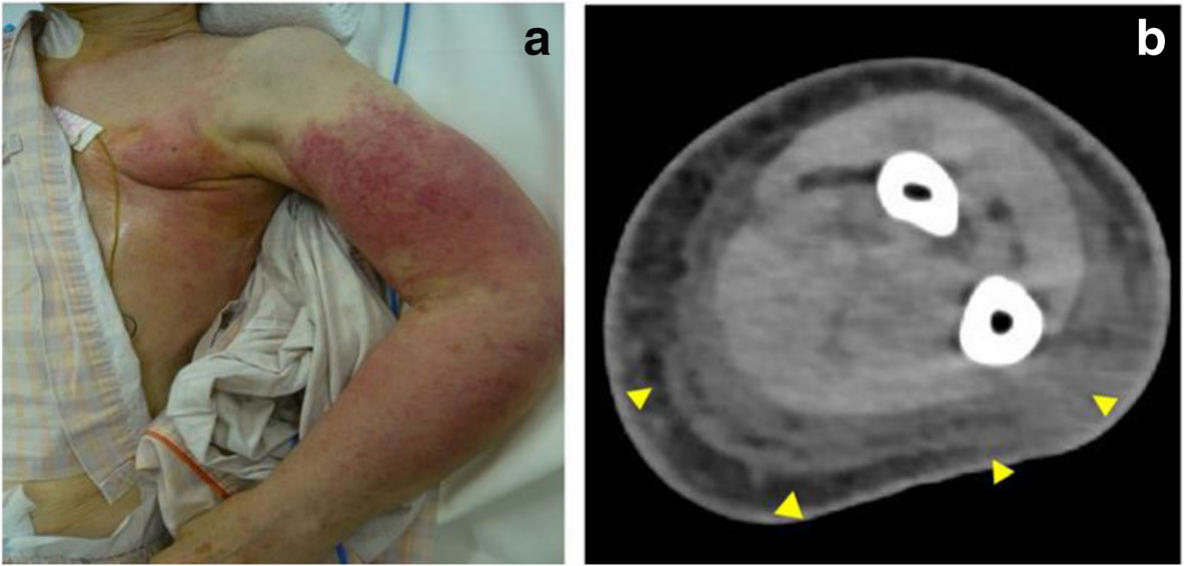
**a** Generalized erythematous macular rash on the left arm. **b** Computed tomography of the left arm, showing thickening of the superficial fascial layer (*triangles*)

She presented to our hospital the day after a high fever and severe pain in her left arm with erythema. She initially reported only slight pain in her left arm, but serious aggravation developed within 24 hours, along with a rash and high fever. She presented with: blood pressure, 90/40 mmHg; heart rate, 120 beats/minute; axillary body temperature, 39.6 °C; respiratory rate, 24 breaths/minute; and peripheral oxygen saturation, 96%. She was in a confused state, and a physical examination revealed swelling of her left upper limb and a generalized erythematous macular rash (Fig. 1a). Laboratory data indicated a high inflammatory state with extreme leukopenia and disseminated intravascular coagulation (DIC): white blood cell count (WBC), 900/μL; platelets (PLT), 7.8×10^4^/μL; international normalized ratio, 1.7; activated partial thromboplastin time, 48.5 seconds; C-reactive protein, 1.3 mg/dL; fibrocyte-derived protein (FDP), 53.7 μg/mL; and procalcitonin, 8.37 ng/mL (Table 1). Computed tomography (CT) of her left arm showed thickening of the superficial fascial layer (Fig. 1b). There were no remarkable changes in chest X-rays. Severe soft tissue infection of her left upper limb with septic shock and DIC was diagnosed. As a result, imipenem/cilastatin (IMP/CS) at 3.0 g/day, intravenously administered immunoglobulin (IVIG) at 5.0 g/day, thrombomodulin at 12,800 U/day, noradrenaline at 0.1 μg/kg per minute, and 30% oxygen were immediately administered. Her hemodynamic status stabilized with the administration of noradrenaline, and her mean arterial pressure value was greater than 65 mmHg, which is recommended by the Surviving Sepsis Campaign.

**Table 1:** Laboratory data at hospital admission

WBCs	900	/μL	Total protein	5.4	g/dL
Metamyelocytes	6	%	Albumin	3.6	g/dL
Band neutrophils	37	%	Na	137	mEq/L
Segmental neutrophils	26	%	K	3.5	mEq/L
Lymphocytes	25	%	Cl	109	mEq/L
Hemoglobin	11.5	g/dL	PT-INR	1.7	
Platelets	7.8	×10^4^/μL	APTT	48.5	sec
			FDP	53.7	μg/mL
Procalcitonin	8.37	ng/mL			
CRP	1.3	mg/dL			
			Arterial blood gas^a^		
CK	92	IU/L	pH	7.46	
LDH	200	U/L	PaCO_2_	30.1	mmHg
AST	24	IU/L	PaO_2_	124	mmHg
ALT	13	IU/L	HCO_3_^-^	21	mmol/L
Total bilirubin	0.8	mg/dL	BE	−1.7	mmol/L
Blood urea nitrogen	20	mg/dL	Lactate	2.4	mmol/L
Creatinine	0.61	mg/dL	Glucose	156	mg/dL

^a^Arterial blood gas was obtained at FiO_2_ 30%

*ALT* alanine aminotransferase, *APTT* activated partial thromboplastin time, *AST* aspartate aminotransferase, *BE* base excess, *CK* creatine kinase, *CRP* C-reactive protein, *FDP* fibrocyte-derived protein, *FiO*_*2*_ fraction of inspired oxygen, *HCO*_*3*_^*-*^  hydrogencarbonate, *LDH* lactate dehydrogenase, *PaCO*_*2*_ partial pressure of carbon dioxide in arterial blood, *PaO*_*2*_ partial pressure of oxygen in arterial blood, *PT-INR* prothrombin time/international normalized ratio, *WBC* white blood cell count

**Fig. 2:**
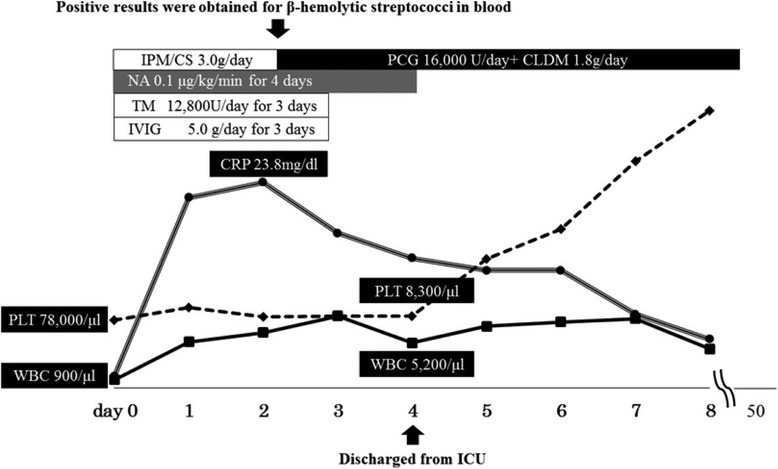
White blood cell counts, platelet counts, C-reactive protein concentration in the peripheral blood, and medication administration. *CLDM* clindamycin, *CRP* C-reactive protein, *ICU* intensive care unit, *IPM/CS* imipenem/cilastatin, *IVIG* intravenously administered immunoglobulin, *NA* noradrenaline, *PCG* penicillin G, *PLT* platelet, *TM* thrombomodulin, *WBC* white blood cell

Two days after starting this therapy, her C-reactive protein was still elevated at 23.8 mg/dL, and positive results for Gram-positive cocci, probably *Streptococcus*, were obtained from the first cultures. Aerobic and anaerobic blood cultures were taken at each assessment. Culture of the cellulitis was not taken. Antibiotics were changed to penicillin G (PCG) at 16,000 U and clindamycin (CLDM) at 1800 mg/day. Group G SDSE was later identified using the Lancefield grouping and an automated bacterial identification system (VITEK® 2; bioMerieux, USA), which showed category agreement from 96 to 100% for *Streptococcus agalactiae* and from 91 to 100% for *Streptococcus pneumoniae* in antimicrobial susceptibility testing [3]. The isolated SDSE was highly sensitive to PCG and CLDM. The minimum inhibitory concentrations (MICs) were under 0.06 μg/ml for PCG and under 0.12 μg/ml for CLDM. Administration of IVIG and thrombomodulin were continued until day 3. Four days after starting treatment, PLT counts indicated a tendency toward improvement, and noradrenaline was completely withdrawn (Fig. 2). Her left arm was still edematous, but the severe pain had resolved. She was discharged from our intensive care unit (ICU) on day 4. Two sets of blood cultures, which were collected 7 days after starting the treatment, were both negative, and intravenous administration of antibiotics (IMP/CS, PCG with CLDM) was continued until 18 days after starting the therapy. During the following 2 weeks, oral administration of 900 mg per day of CLDM was prescribed.

She came to our out-patient clinic every 3 months for 2 years. There was no recurrence of breast cancer or cellulitis within 2 years of the onset of streptococcal toxic-shock syndrome (STSS).

## Discussion

Breast cancer is one of the most common cancers in women, with an incidence rate of 89.7 per 100,000 women in Western Europe. High survival rates have been achieved in these countries [1], but these high survival rates can be accompanied by other problems. BCRL is one of the most common complications after breast cancer treatment. The overall incidence of BCRL has been reported as 26% after mastectomy, and axillary lymph node dissection with axillary radiotherapy significantly increases the risk of BCRL (relative risk, 6.9) [4, 5]. As lymphedema provides an ideal environment for bacterial growth, probably because of the decreased lymphatic flow and impaired elimination of phagocytosed bacteria, soft tissue infection is quite common as a complication among patients with BCRL [6]. Most of the causative pathogens are *Staphylococci* or *Streptococci*, and infection is often treated without serious progression. However, streptococcal infections occasionally develop into STSS. The development of STSS significantly increases the risk of mortality, which may exceed 50% [7]. Criteria for the diagnosis of STSS were established by the Centers for Disease Control and Prevention (CDC) in 2010 (Table 2) [8]. Most cases of STSS are caused by GAS, but cases caused by SDSE belonging to Lancefield serogroup C or G have recently been reported. In 2011, a Japanese surveillance study reported the involvement of GAS in 78% of STSS (76 cases), group G streptococci in 21% (22 cases, all involving SDSE), and group C streptococci in less than 2% (two cases, one with SDSE, and the other with *Streptococcus constellatus* *subspecies* *pharyngis*) [9]. Although GAS can affect even the healthy, SDSE mainly affects elderly individuals with chronic diseases, such as diabetes mellitus, cardiovascular disease, malignancy, and immunosuppression. In particular, breakdown of the skin was noted in 30 to 60% of cases [2], while the most common clinical manifestation of invasive SDSE infection is cellulitis (41%) [2]. The mortality rate from SDSE bacteremia is 15 to 18%, which is comparable to that for GAS bacteremia. SDSE infection by itself is considered less severe than GAS bacteremia, but the effects of patient characteristics, such as age and underlying diseases, may increase the infection severity [2].

**Table 2:** Clinical criteria for the diagnosis of streptococcal toxic-shock syndrome

1. Hypotension	
2. Multi-organ involvement characterized by two or more of the following:	
Renal impairment	
Coagulopathy	
Liver involvement	
Acute respiratory distress syndrome	
A generalized erythematous macular rash that may desquamate	
Soft-tissue necrosis, including necrotizing fasciitis or myositis and gangrene	
Isolation of *Streptococcus* from	
Non-sterile site ⇒ Probable	
Normally sterile site ⇒ Confirmed	

Streptococcal toxic-shock syndrome Case Definition 2010 [8]

In our case, the patient had undergone left mastectomy and axial lymph node dissection with additional chemotherapy and radiotherapy following hormone therapy. Under such aggressive treatment, no sign of recurrence was seen for more than 2 years postoperatively, despite advanced lymph node metastasis. However, mastectomy with axial dissection and radiotherapy induced BCRL. This elderly woman with BCRL was at high risk of invasive SDSE infection and eventually developed STSS induced by SDSE. The clinical course, which progressed rapidly (within 24 hours) and emerged in the soft tissue, was typical of STSS [7]. The route of transmission of the pathogen into the deep soft tissue was uncertain, but the edematous subcutaneous tissue might have provided a suitable environment for exponential bacterial growth. She presented signs of septic shock, requiring noradrenaline for 4 days in our ICU. In addition, her confused mental state, soft tissue infection following DIC with high inflammatory status (CRP 23.8 mg/dL; procalcitonin 8.37 ng/mL) and extreme leukopenia (900/μL) all suggested severe infection and toxicity. Thrombocytopenia with elevation of FDP also remained until day 4, despite appropriate DIC therapy (Fig. 2). Lastly, SDSE was isolated from the blood, and STSS was confirmed based on the CDC criteria (Table 2), with DIC, soft tissue infection, and generalized erythematous macular rash. SDSE is not as toxic as GAS, and no vital organ dysfunction developed, such as respiratory failure, liver involvement, or renal impairment. These factors were considered the main reasons why our patient survived her case of STSS. This report highlights the risk of life-threatening infection developing among survivors of malignancy. Therefore, invasive streptococcal infection should be taken into account as a risk factor after breast cancer treatment. Ageing and advances in medicine have created the need to address such cases.

## Conclusion

We have reported a case of SDSE-induced STSS with BCRL. Infectious diseases can become a life-threatening problem after successful treatment for malignant disease.

## Abbreviations

BCRL: Breast cancer-related lymphedema
CDC: Centers for Disease Control and Prevention
CLDM: Clindamycin
DIC: Disseminated intravascular coagulation
FDP: Fibrocyte-derived protein
GAS: Group A streptococci
ICU: Intensive care unit
IMP/CS: Imipenem/cilastatin
IVIG: Intravenously administered immunoglobulin
PCG: Penicillin G
PLT: Platelets
SDSE: *Streptococcus dysgalactiae* *subspecies* *equisimilis*
STSS: Streptococcal toxic-shock syndrome
